# Novel Production of Bovine Papillomavirus Pseudovirions in Tobacco Plants

**DOI:** 10.3390/pathogens9120996

**Published:** 2020-11-28

**Authors:** Inge Pietersen, Albertha van Zyl, Edward Rybicki, Inga Hitzeroth

**Affiliations:** 1 Biopharming Research Unit, Department of Molecular and Cell Biology, University of Cape Town, Cape Town 7701, South Africa; inge.pietersen@alumni.uct.ac.za (I.P.); alta.vanzyl@uct.ac.za (A.v.Z.); ed.rybicki@uct.ac.za (E.R.); 2 Institute of Infectious Disease and Molecular Medicine, University of Cape Town, Cape Town 7701, South Africa

**Keywords:** pseudovirion, BPV, biopharming

## Abstract

Vaccine efficacy requires the production of neutralising antibodies which offer protection against the native virus. The current gold standard for determining the presence of neutralising antibodies is the pseudovirion-based neutralisation assay (PBNA). PBNAs utilise pseudovirions (PsVs), structures which mimic native virus capsids, but contain non-viral nucleic material. PsVs are currently produced in expensive cell culture systems, which limits their production, yet plant expression systems may offer cheaper, safer alternatives. Our aim was to determine whether plants could be used for the production of functional PsVs of bovine papillomavirus 1 (BPV1), an important causative agent of economically damaging bovine papillomas in cattle and equine sarcoids in horses and wild equids. BPV1 capsid proteins, L1 and L2, and a self-replicating reporter plasmid were transiently expressed in *Nicotiana benthamiana* to produce virus-like particles (VLPs) and PsVs. Strategies to enhance particle yields were investigated and optimised protocols were established. The PsVs’ ability to infect mammalian cells and express their encapsidated reporter genes in vitro was confirmed, and their functionality as reagents in PBNAs was demonstrated through their neutralisation by several different antibodies. This is the first report of BPV PsVs expressed in plants and demonstrates the potential for the development of therapeutic veterinary vaccines in planta.

## 1. Introduction

Bovine papillomaviruses (BPVs) are a group of epitheliotropic viruses which are of significant importance, both as etiological agents of veterinary diseases and as long-standing models for the study of human and other papillomaviruses (PVs) [1]. Of these, the delta-BPVs (and BPV1 and BPV2 in particular) are the most prevalent, important, and interesting due to their ability to infect and cause disease in a wide range of non-host animals, including horses [2,3], sheep [4,5], domestic and wild cats [6,7], and wild ungulates such as zebra, giraffes, antelope [8,9], and buffalo [8,10,11,12]. Diseases caused by BPVs have large economic impacts, as animals are often prematurely culled due to their physical and functional deterioration, and to prevent the spread of disease to whole herds [13]. Equine sarcoids of horses and wild animals are also of particular importance due to the high costs and ecological value of these animals, and because current treatments are largely ineffective [14,15]. Autogenous vaccines are still most commonly used for prophylaxis, and while a growing body of evidence indicates that these vaccines have some therapeutic effects in cattle [16,17,18] and equids [8,9,19], these vaccines also carry risks of reversion/recombination and exposing the animal to blood-borne diseases [20]. They are further limited by the fact that they require large amounts of wart tissue and a skilled technician to produce the vaccine, only provide protection against particular local strains, and are not DIVA (differentiating infected from vaccinated animals) compliant, thus hindering the serological surveillance of diseases [21]. In recent years, however, virus-like particle (VLP)-based vaccines have emerged as alternatives to traditional attenuated or autogenous vaccines. These vaccines are a class of subunit vaccines, which consist of one or more structural proteins that self-assemble into structures which mimic, but lack, genomic DNA of the native virus [22,23]. VLP-based vaccines have advantages such as enhanced safety and DIVA-compliance, and because they are structurally similar to native virions, are highly immunogenic and present the repetitive conformational epitopes of the native virus, which activate B-cells by binding to their associated immunoglobulin receptors. Because they are also processed and presented by major histocompatibility complex class II (MHC-II) molecules for T-helper cell activation, VLPs elicit both humoral and cell-mediated responses [20]. Several VLP-based and capsid subunit vaccine candidates against BPV types 1 [24,25], 2 [23,26], and 4 [22,23,27] have been produced and have been shown to confer long-lasting and efficient protection against BPV challenge yet, in spite of these successes, there is currently no widespread BPV vaccine available commercially [20,21,28]. This is largely due to the costs associated with the cell-based production platforms currently required for VLP production, which has limited the widespread application of VLP-based vaccines, especially in veterinary settings where low vaccine costs are essential to encourage widespread vaccination [1,28]. In addition to their use as prophylactic vaccines, VLP-based particles which contain non-viral nucleic acid pseudogenomes, known as pseudovirions (PsVs), can be used as surrogates for native viruses in biological studies, as vectors for the in vivo delivery of DNA vaccines into specific cells for gene expression and targeted therapy [29,30,31,32], or as reagents in PsV-based neutralisation assays (PBNAs) [32,33,34]. PBNAs are used to determine the presence of virus-specific neutralising antibodies (NAbs) in antisera following natural infection or immunisation, and are an essential part of epidemiological studies and vaccine development [34]. For these assays, the pseudogenomes of the PsVs usually contain reporter genes, which are expressed upon their infection of cell cultures and can be used to indicate their infectivity or to measure the degree to which neutralising antibodies bind to epitopes on the surface of the PsVs and prevent their infection of cells [35]. Because PBNAs are an independently conducted, unbiased means of assessing the performance of vaccines through the presence of protective antibodies, these assays are considered the gold standard for vaccine evaluations, and have been extensively used in studies of human papillomaviruses (HPVs) [36,37,38,39,40,41,42,43] and BPVs [26,44]. As with VLPs, PsVs are mainly produced in expensive cell cultures which has limited their widespread application, yet plant-expression platforms could potentially mitigate the production costs and production time, and still serve to maintain the conformational integrity of the antigens through plants’ ability to affect the post translational modifications required for protein folding and stability [45,46]. Several studies have demonstrated that plants can be used to produce functional PV VLPs and PsVs [47,48,49,50,51], including one in which small BPV1 VLPs were produced in *Nicotiana benthamiana* and elicited strong immunogenic responses in rabbits [47]. In this study, we explored whether methods developed for transient expression of HPV16 PsVs in *N. benthamiana* could be applied more broadly to express BPV1 PsVs, and whether these could be used as reagents in PBNAs.

## 2. Results

### 2.1. Transient Expression of BPV1 VLPs in N. benthamiana

**Figure 1:**
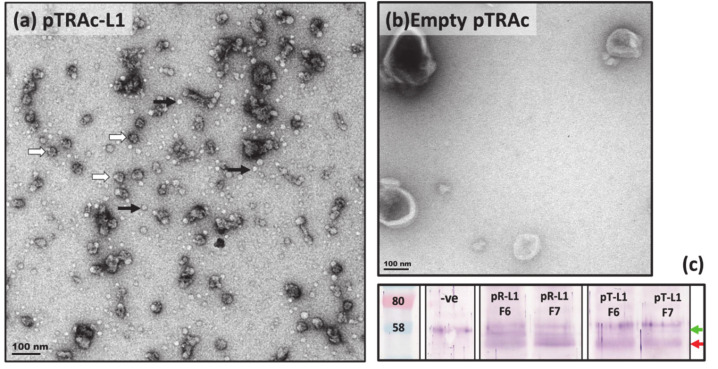
Transmission electron micrographs and western blot of purified plant-produced bovine papillomavirus 1 (BPV1) virus-like particles (VLPs) and controls. (**a**) BPV1 VLPs obtained from pTRAc-L1 infiltrated plants harvested at 5 days post infiltration (dpi). Proteins were purified by ultracentrifugation through a 27–50% iodixanol (OptiPrep^TM^) density gradient. Purified fractions (F1–F13) were analysed by dot blot probed with anti-L1 antibody (data not shown) and fractions with the highest L1 signal were negatively stained with uranyl acetate and visualised under TEM (scale bar 100 nm for both micrographs). Capsomeres (~10 nm) are indicated by grey arrows and *T* = 1 VLPs (~30 nm) are indicated by white arrows. (**b**) pTRAc empty vector control in which no higher order structures were observed (**c**) Western blots of purified pTRAc-empty negative control (-ve), and pRIC3.0-L1 (pR-L1) and pTRAc-L1 (pT-L1) fractions F6 and F7, probed with “Abcam” (#ab2417) BPV-1/1H8+ CAMVIR antibody (1:1000). BPV1 L1 is indicated with the red arrow at ~52 kDa and an unidentified plant protein is indicated in green at ~58 kDa.

To first establish that BPV1 capsid proteins expressed in *N. benthamiana* were able to self-assemble into higher order structures and survive purification with techniques developed for HPVs [50], we performed infiltrations of L1-only and L1 + L2 for the production of VLPs. For this, genes encoding for BPV1 capsid proteins, L1 and L2, were codon-optimised for expression in *N. benthamiana*, as optimisation for tobacco was shown in previous studies to increase protein expression [47]. The genes were subcloned into the pRIC3.0 and pTRAc plant expression vectors, and the constructs were agroinfiltrated into *N. benthamiana* plants. Biomass was harvested at 5 days post infiltration (dpi) and proteins were purified using methods described in Lamprecht et al. (2016) [50]. Briefly, protein extracts from processed biomass were concentrated on sucrose cushions by ultracentrifugation, then separated on discontinuous iodixanol gradients prepared in a high salt phosphate-buffered saline (1× HSPBS) buffer. Gradients were fractionated into 1 mL fractions from the bottom of the tube, and protein expression and particle assembly were established by dot blot, western blot, Coomassie-stained polyacrylamide (PA) gels, and transmission electron microscopy (TEM) analyses (Figure 1). 

Western blots of purified protein fractions probed with anti-BPV1-L1 antibodies showed bands of ~52 and ~58 kDa in both the L1 and L1/L2 samples, yet the ~52 kDa band was absent from empty vector negative controls (Figure 1c). These results indicate that the 52 kDa band corresponds with plant-expressed BPV1 L1, for which the native protein has an expected size of ~55 kDa [25], and the presence of L1 in this band was confirmed with mass spectrometry (data not shown). TEM analyses were performed on purified fractions with the highest BPV1 L1 signal, as determined in dot and western blots. Micrographs showed the presence of spherical particles of 25–30 nm in diameter in both the pTRAc-L1 and pRIC3.0-L1 purifications, yet none were observed in the empty vector negative controls of pRIC3.0 (data not shown) or pTRAc (Figure 1b). These findings indicate that the ~30 nm particles observed were *T* = 1 BPV1 VLPs, similar to those obtained in other plant expression studies of PVs [47,51], and assembly intermediates such as capsomeres (pentameres) of ~10 nm were also observed [52,53]. These findings showed that plant-expressed BPV1 L1 was capable of assembling into higher order structures, and that these structures were maintained throughout the purification process. Few observable differences were seen in the size and number of VLPs obtained by L1 expression with pRIC3.0 and pTRAc (Supplementary Figure S1), and similar but fewer VLPs were observed in the L1/L2 co-expression studies (Supplementary Figure S2). Previous studies have shown that L2 facilitates encapsidation of the viral genome, and that neither mammalian [32] nor plant-produced (R. Lamprecht 2017, personal communication) can be produced without the presence of both L1 and L2. The successful formation of PsVs in subsequent studies thus confirmed that both L1 and L2 were expressed and incorporated into these particles.

### 2.2. Expression and Purification of Plant-Produced BPV1 PsVs

For the production of BPV1 PsVs, plants were co-infiltrated with L1, L2, and pRIC3.0-mSEAP, a self-replicating construct with a mammalian expression cassette encoding a secreted embryonic alkaline phosphatase (SEAP) protein. Plant biomass was harvested 4 dpi and purifications were performed using methods established for HPV PsV production, and described previously for VLPs [50]. However, in the present purifications, the concentration of the high salt buffer (1× HSPBS) used to prepare the final density gradient was increased 6-fold (i.e., 6× HSPBS = 6× PBS + 3M NaCl). Fractions of purified proteins were analysed by dot blot, western blot, Coomassie-stained PA gels, and TEM.

**Figure 2:**
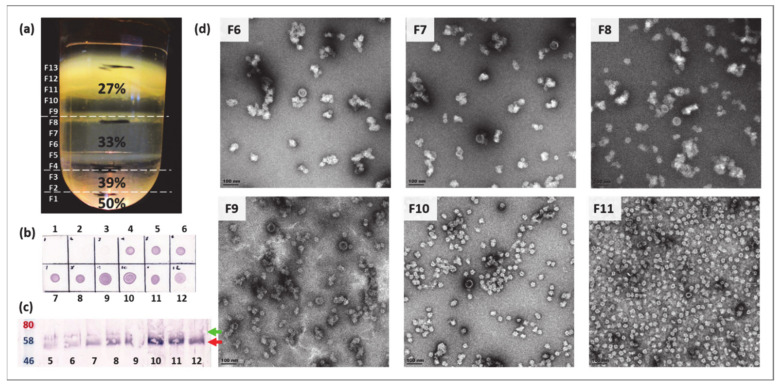
Plant-produced purified BPV1 pseudovirions (PsVs) fractions. (**a**) Discontinuous iodixanol (OptiPrep™) density gradient of concentrated BPV1 proteins after centrifugation. Concentrations of iodixanol are indicated as percentages (%) and the 1 mL fractions into which they were collected are indicated as F1–F13. Opaque bands at the bottom of 33% and 27%, and the top of 27% indicate where L1 was concentrated. (**b**) Dot blot of fractions F1–F12 and (**c**) Western blot of F5–F12. Both blots were probed with Dako anti-BPV1-L1 antibody (1:1000). BPV1 L1 is indicated by a red arrow (~55 kDa) and plant protein (~60 kDa) is indicated by a green arrow. (**d**) Representative TEM images of fractions in which the greatest numbers of higher order structures were observed (F6–F11). Scale bars represent 100 nm.

**Figure 3:**
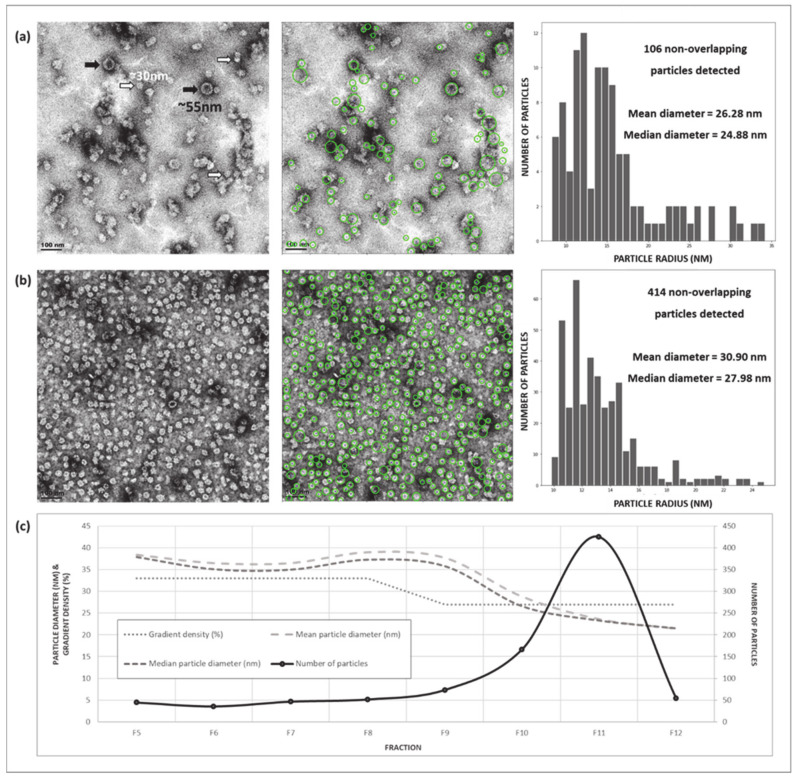
Transmission electron micrographs (TEM) and particle analysis of pseudovirion protein purifications. TEM images and corresponding size distribution histograms of (**a**) Fraction 9 (F9), from the bottom of the 27% iodixanol (OptiPrep^TM^) gradient, in which the greatest number of 50–60 nm *T* = 7 particles (PsVs) was seen (indicated by black arrows); and (**b**) Fraction 11 (F11) from the upper 27% gradient, in which the smaller ~30 nm *T* = 1 VLPs (white arrows) predominated and few larger particles were observed. The scale bar is 100 nm for all images. Size distribution histograms were created using a custom Python script which identified, measured, and counted non-overlapping spherical objects (circled in green). (**c**) Line graph depicting the size and quantity (as determined by the Python script) of particles distributed throughout the density gradient.

Visual inspection of the discontinuous iodixanol (OptiPrep™, Merck, Kenilworth, NJ, USA) density gradient revealed three distinct opaque bands, present at the bottom of the 33%, the bottom of the 27%, and the upper fractions of the 27% gradients (Figure 2a). Dot and western blot analyses (Figure 2b,c) revealed the presence of L1 at ~55 kDa (previously observed at 52 kDa, Figure 1c), while the plant protein was observed as a band of 60 kDa and was mostly present in the lower fractions (F4–F8). The western blot indicated that L1 was present in all fractions from F5 (33%, ~1.30 mg/mL) upwards, and that the largest amount of L1 was present in the upper (less dense) fractions, F7–F12, indicating that particulate proteins of different densities and concentrations were present across the gradient. The presence of the plant and L1 proteins in their respective fractions correspond with the opaque bands observed in the density gradient. Faint bands of L1 (~55 kDa) were visible on Coomassie-stained PA gels (data not shown) indicating relatively low overall yields of protein. TEM analyses were performed on fractions with the strongest L1 signal on western blots, to determine whether particles were successfully assembled. Electron micrograph imaging confirmed the presence of particles of ranging from 25 to 30 nm in diameter and, in F6 and higher, 50–60 nm *T* = 7 structures, which closely resemble the *T* = 7 structure of native BPV virions [1,53] and those of BPV1 PsVs produced in mammalian cells [54]. No capsomeres (10 nm) were observed in any of the fractions, yet aggregates of protein and partially formed capsids of 40–50 nm (indicative of incomplete particle assembly) were present in various fractions (Figure 2d and Supplementary Figure S3). To quantitatively assess particle sizes and numbers, TEM micrographs of F5–F12 were analysed by a custom Python script (https://github.com/CorrieGunter/particle_counter) [55], which identified, measured, and counted non-overlapping spherical objects (Figure 3).

The data from the Python script were depicted in histograms and a line graph, (Figure 3) which showed that 25–30 nm particles predominated throughout the density gradient, but were most abundant in F11 of the 27% iodixanol gradient (~1.28 g/mL), while larger particles were present mostly in the 33% iodixanol fractions (~1.30 g/mL).

### 2.3. Infectivity Assays of Plant-Produced BPV PsVs

To determine whether functional PsVs had formed, and in which the largest numbers were present, diluted protein fractions were incubated with mammalian HEK293 TT cells. The cells were assayed for the presence of the SEAP reporter protein to confirm that the infection and expression of the reporter construct had occurred.

**Figure 4:**
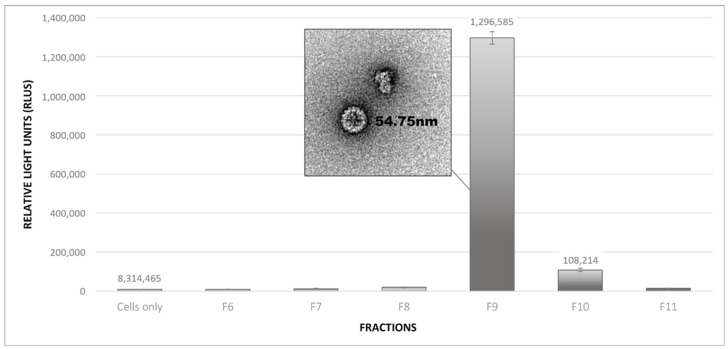
Bar graph of relative SEAP expression in HEK293TT by pseudoinfection of plant-produced BPV1 PsVs. A bar graph of chemiluminescence readings (as relative light units—RLUs), indicating the amount of secreted alkaline phosphatase (SEAP) protein expressed by the HEK293TT cells upon infection with 1:10 dilutions of BPV1 protein fractions. A negative control of cells to which no BPV1 proteins had been applied was included as a baseline reading for the background signal. Error bars of two readings per sample are indicated. The highest SEAP readings (~1.3 × 10^6^ RLUs) were obtained for fraction F9, which corresponds with the TEM micrographs in which the greatest number of ~55 nm, *T* = 7 particles (an example of which is shown in the micrograph) was observed.

Levels of SEAP expression were measured by chemiluminescence as relative light units (RLUs), and readings showed that fraction 9 (F9) from the bottom of the 27% density gradient had the highest SEAP signal (~1.3 × 10^6^), 150-fold higher than the cells-only baseline (Figure 4). The next highest was F10, which had a reading 13-fold higher than the baseline, yet 12-fold lower than F9. These readings correspond with fractions analysed by TEM, in which the greatest number of *T* = 7 particles was observed, and confirmed that BPV1 PsVs were successfully produced in planta. However, the presence of *T* = 7 particles in fractions F6–F8, for which low SEAP readings were obtained, suggested that not all of the *T* = 7 structures contained plasmid DNA.

### 2.4. Purification and Expression Optimisation of BPV VLPs and PsVs

To increase protein and particle yields, various purification and expression strategies were explored, and the protocol established for HPV PsV production was optimised to increase the production yields of BPV PsVs. Optimisation studies were carried out on the same batches of plants for direct comparisons in order to reduce the influence of batch-to-batch variability often encountered in plant expression studies.

#### 2.4.1. Effect of Biomass Freezing on Particle Stability

As it is often impractical to process biomass immediately after harvesting, freezing of plant biomass at −80 °C for later processing is a common practice, and was assessed in this study. A visual comparison of TEM images of BPV1 PsV purified from freshly processed biomass versus biomass that had been frozen at −80 °C and processed several weeks later indicated that freezing had no detrimental effect on particle stability (data not shown), and all subsequent purifications were performed using frozen biomass. Later observations suggested that particle yields from frozen biomass might even be higher, possibly due to the freeze–thaw rupturing of cells releasing greater numbers of particles, yet this hypothesis was never tested.

#### 2.4.2. Optimisation of Density Gradient Ultracentrifugation

In the original protocol, a 30% sucrose gradient layered on top of a 50% sucrose cushion is used to concentrate PsVs into the 30% fraction. However, TEM analysis of the 50% cushion and pellet at the bottom of the tube, both of which are usually discarded, revealed the presence of *T* = 7 particles which had passed into and through the 50% sucrose cushion instead of remaining suspended in the 30% sucrose gradient. Iodixanol was therefore explored as an alternative density medium and, as no particles were observed in TEM images of the 50% iodixanol cushion or pellet, was adopted for all further cushion gradients (data not shown). 

Initial results of BPV PsVs purifications suggested that the use of a high salt concentration in the final density gradient might have a positive influence on the number and quality of particles obtained (Figure 2), and a comparison of density gradients prepared with the 1× HSPBS buffer specified in the original protocol versus a 6× concentration of the same buffer was performed. TEM analyses showed that fewer aggregates were present in the 6× HSPBS gradient, and that the particles (VLPs and PsVs) formed appeared to be more structured (Supplementary Figure S4). A SEAP assay revealed that PsVs were concentrated in fewer fractions than those of the 1× HSPBS gradient (Supplementary Figure S5). These outcomes are probably a result of the increased osmotic pressure associated with higher salt concentrations, though interaction mechanisms and their effects on proteins and particles were not elucidated. One hypothesis is that the increased density of the medium compressed the particles by forcing proteins together, enhancing their structures, and that in creating gradients of higher densities through which particles have to migrate, an enhanced separational effect may have occurred, causing particles with particular properties (densities) to be concentrated together.

#### 2.4.3. Expression Studies: Heat Shock Treatment, Increased Acetosyringone, and Extended in Planta Maturation

Strategies to increase the expression of recombinant protein and enhance PsV yields were also explored, and were tested and compared on a single batch of plants. Methods investigated were based on observations in purification studies, and on methods described by Norkunas et al. (2018) [56], and included: (i) increasing the acetosyringone concentration to enhance transfer of genetic material into the host cells; (ii) exposing plants to a brief heat shock treatment at 2 dpi; and (iii) extending the incubation time of plants from 4 to 6 dpi before harvesting biomass. Biomass was purified using the optimised purification strategy developed in this study, and fractions were analysed by western blot and TEM analysis. 

**Figure 5:**
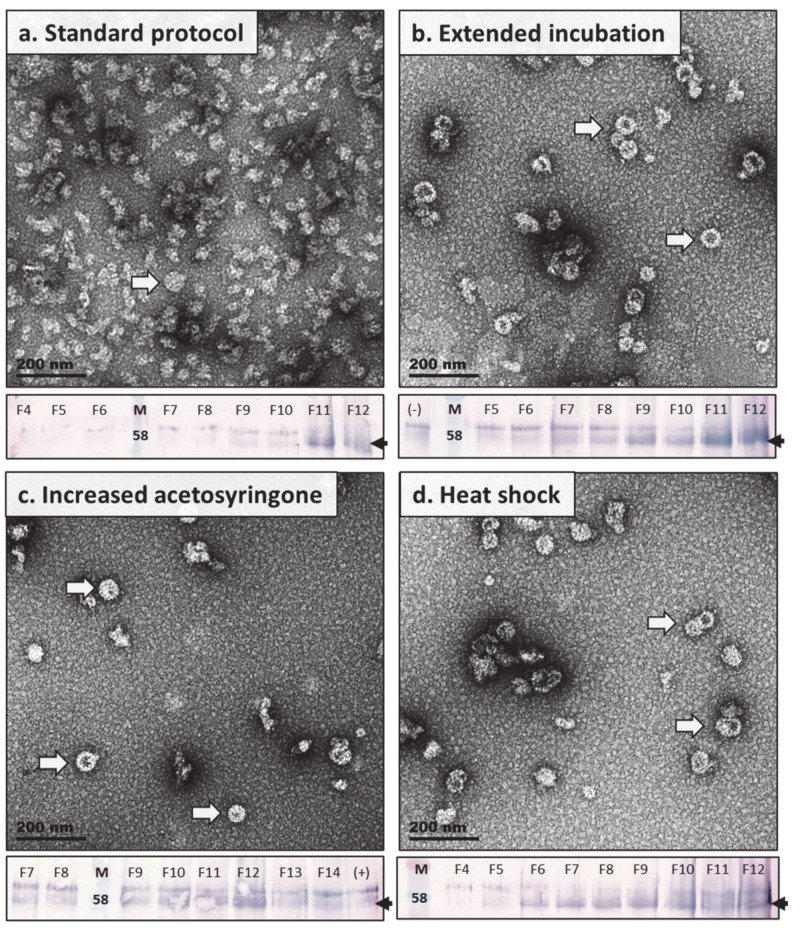
Transmission electron micrographs and western blots of BPV1 PsV expression optimisation studies. Representative images of fractions with the greatest number of putative (50–60 nm, *T* = 7) PsV particles, indicated by white arrows, are shown for the following expression conditions: (**a**) Standard protocol, as established for HPV16 PsV expression; (**b**) Extended in planta incubation (harvested 6 dpi); (**c**) Increased acetosyringone (500 μM); (**d**) Heat shock treatment (37 °C). Magnification was performed at 53,000× and scale bars represent 200 nm. Western blots of the listed expression conditions are shown below their corresponding micrographs. Blots were probed with Dako anti-BPV1-L1 (1:1000) and the L1 protein is indicated by black arrows at ~55 kDa.

Western blot analyses (Figure 5) suggested that levels of L1 accumulation for PsVs expressed by the standard method developed for HPV were lower than those obtained by extending the in planta incubation period, increasing the concentration of acetosyringone used for *Agrobacterium* induction, or applying a heat shock treatment to the plants. However, it appeared that the western blot of the standard method had not transferred properly, as stronger bands either of L1 or plant proteins would be expected from the high levels of loose protein aggregates and immature VLPs and PsVs observed in TEM (Figure 5a and Supplementary Figure S6). TEM analyses, however, revealed that the highest numbers of, and most well-structured/matured, PsVs and VLPs were observed in modified expression methods. It appeared, from the micrographs, that extending the incubation time for protein expression in plants by 2 days (harvest 6 vs. 4 dpi) yielded the greatest number of higher order particles (Figure 5b) followed by those obtained by heat shock treatment (Figure 5c). Increasing acetosyringone concentrations also increased particles numbers but not by as much as the aforementioned methods, yet particles produced by this method did appear the most structured of the expression strategies explored (Figure 5d).

### 2.5. Pseudovirion-Based Neutralisation Assay of Plant-Produced BPV1 PsVs

**Figure 6:**
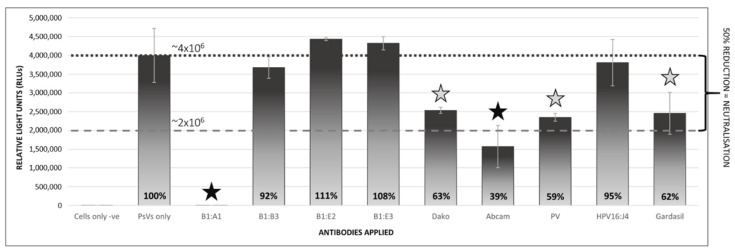
Pseudovirion-based neutralisation assay (PBNA) of plant-produced BPV1 PsVs. Bar graph showing SEAP assay readings (RLUs) of plant-produced BPV1 PsVs which were pre-incubated with a set of papillomavirus antibodies and used to infect HEK293TT cells. The antibodies applied to the PsVs at a 1:200 dilution included antibodies against BPV (B1:A1, B1:B3; B1:E2; B1:E3, Dako, Abcam), HPV (Gardasil, Abcam, HPV16:J4) and an unspecified PV. Baseline readings were established from a negative control of uninfected cells (“Cells only”). The degree of neutralisation was determined against the mean readings of cells infected with PsVs to which no antibodies had been applied (“PsVs only”). These readings were set at 100%. Black stars indicate complete neutralisation (>50% reduction in SEAP readings) and grey stars indicate partial neutralisation (50–75% neutralisation).

The production of NAbs against specific foreign antigens by the humoral immune system is the most important factor in the prevention of infection by PVs. These antibodies target immunodominant epitopes, and vaccine antigens are therefore selected on the basis of their immunogenicity [57]. To assess their antigenicity and whether they could be used as reagents to evaluate antisera in PBNAs, the plant-produced BPV PsVs were assayed for neutralisation with an assortment of PV antibodies, namely, B1:A1, B1:B3, B1:E2, B1:E3, Dako, Abcam, PV, HPV16:J4, and Gardasil. The PsVs were diluted 1:10 and antibodies were applied at concentrations of 1:200, and the mixture was incubated for 1 h at 4 °C before being applied to the cells. Cells were incubated for 3 days, and SEAP expression was measured to determine the neutralising potential of the antibodies, through a reduction (of at least 50%) in SEAP signal (Figure 6). Reactions were performed in duplicate, and the PBNA was repeated with a different set of PsV stock, to which antibodies were applied at 1:500.

At antibody concentrations of 1:200, both the Abcam and B1:A1 antibodies showed a reduction of over 50% in the SEAP signal, yet only B1:A1 was fully neutralising (100%) at both 1:200 and a further dilution of 1:500 (data not shown). The Dako, PV, and Gardasil antibodies proved to be partially neutralising at higher antibody concentrations, which could indicate that these bind to fewer epitopes on the PsVs than do the B1:A1 antibodies, are non-conformational antibodies, or do not bind to neutralising epitopes on the PsVs that would limit or prevent their attachment and entry into cells. This is unsurprising, as both the Abcam and Dako antibodies were produced against chemically disrupted BPV1 virions for which conformational epitopes were likely lost and, as the Gardasil antibody was prepared against the HPV6, −11,−16, and −18 VLP-based Gardasil vaccine, it would share only a few epitopes with BPV1.

## 3. Discussion

Neutralisation assays, such as PBNAs, are essential to vaccine development and epidemiological studies, as they are used to establish whether virus-specific NAbs were generated after immunisation with the vaccine candidate, or after natural infection with the live virus [24,35,58]. As L1 is the immunodominant protein in the PV viral capsid and elicits higher titres of NAbs than other viral proteins, it has been the main focus of PV vaccinology studies [59]. However, L1-based vaccines are highly specific and their effects are limited to only the viral types included in the vaccine (with the rare exception of BPV1 and 2, which are closely related serotypes capable of some cross-neutralisation of one another [26]), whereas L2 antibodies have been shown to neutralise a range of different PV types within the same species [34], and low-titre antibodies against L2 alone have been shown to neutralise PsVs in vitro [60]. Furthermore, L2-based BPV vaccines have been shown to exhibit therapeutic effects such as early tumour rejection [61], and could enhance efficacy or overcome some of the limitations of L1-only vaccination. In this study, BPV VLPs and PsVs incorporating both L1 and L2 were generated in *N. benthamiana* plants and assessed to determine whether plant systems could be used to generate sufficient quantities of these particles as reagents for vaccine studies or as potential vaccines themselves.

Western blot and protein-stained PA gels of purified plant extracts confirmed the expression of L1, and large-scale purifications of L1 and L1 + L2 expression by pRIC3.0 and pTRAc plant expression vectors both yielded populations of *T* = 1 VLPs with ~30 nm diameters. These particles resembled the *T* = 1 VLPs obtained by Love et al. (2012) [47] when BPV1 L1 was expressed in *N. benthamiana*, as well as those obtained by Matic et al. (2012) [51] when HPV16 L1 was expressed in *N. benthamiana*, indicating that these structures were small BPV1 VLPs, an assumption further supported by the lack of any such structures in plant negative controls. Co-infiltrations of L1, L2, and a replicating reporter plasmid resulted in the formation of *T* = 7 PsVs, as seen in TEM micrographs and confirmed by SEAP assays and PBNAs. However, the presence of *T* = 7 particles in the higher density fractions (bottom of the 33% iodixanol, ~1.3 g/mL density gradient) for which low SEAP readings were obtained, suggested that not all of the *T* = 7 structures contained plasmid DNA. These findings would be consistent with those of Buck et al. (2005) [33], who found a significant portion of empty BPV capsids (*T* = 7 VLPs) among their PsV stocks, and also found that capsids lacking encapsidated DNA migrated down the density gradient into the higher-density fractions (~1.25 g/mL). High numbers of *T* = 1 VLPs were also generated during PsV expression, more than the numbers produced by L1 or L1/L2 expression alone. 

The protocols used for the expression and purification of BPV VLP and PsVs were based on methods established for in planta expression of HPV VLPs/PsVs, and support previous findings that suggest methods developed for specific PV species can be applied to PVs more broadly [62]. The purification strategies used in this study are approximately the same as those used for purification of mammalian PsVs in terms of labour and costs [32], yet plants are a much cheaper production platform than cell cultures, require less maintenance, and are less prone to include dangerous contaminants [46,49,63]. Purification optimisation studies indicated that biomass could be stored at −80 °C for at least several months with no yield losses observed over time, and that the same was true for purified protein fractions, although losses in PsV activity were observed following repeated freeze–thaw cycles. Our results also indicated that crude plant extracts should be concentrated on cushions prepared with iodixanol, so as to avoid the pelleting and losses of particles, although intentional pelleting may be explored as a method for separating particles with different properties, such as *T* = 7 particles with and without encapsidated DNA [62]. We observed that proteins separated on discontinuous gradients prepared with 6× HSPBS and 27–50% iodixanol, yielded protein fractions in which particles were more concentrated and appeared more structured. No losses of particles from using the 6× HSPBS buffer were evident in TEM micrographs, although it was unclear from the SEAP assay whether overall yields of PsVs were similar (Supplementary Figure S5). However, there is some utility in enhancing the separation and concentration of the respective particles (*T* = 7 VLPs vs. *T* = 7 PsVs vs. *T* = 1 VLPs) into smaller volumes, and this method was adopted for all further purifications. Results from the expression studies indicated that the greatest numbers of BPV1 PsVs and VLPs were obtained when the incubation period for protein expression and capsid formation in planta was extended to 6 dpi before biomass was harvested, although plants treated with a 30 min heat shock treatment at 37 °C on 2 dpi, and plants infiltrated with Agrobacterium induction media with increased acetosyringone (500 μM) concentrations also resulted in higher yields. Particles produced by increased acetosyringone were the most structured, yet were slightly fewer in number than those attained by extended incubation and heat shock treatment. While PV L1 VLPs have been shown self-assemble in a protein-dependent manner [64], it would appear that L1 levels do not necessarily correlate with PsV formation, as other studies in our lab have shown [65]. It is possible that the additional integration of L2 into pentamers and their assembly into higher order structures around plasmid DNA may require more time, and that rapid increases and high levels of protein accumulation may result in a bottleneck effect. Further extension of the in planta incubation time or other approaches for the maturation of particles prior to purification should be investigated, as immature capsids are more fragile and may be lost during ultracentrifugation [62].

On the whole, relatively high SEAP readings were obtained for many purifications, in spite of the low protein yields observed in the Coomassie-stained PA gels. These results indicate that efficient assembly and encapsidation of the circular reporter DNA into the L1/L2 capsids had occurred, which would be consistent with the findings of promiscuous packaging observed in the production of PV PsVs in mammalian cell cultures [33]. Our findings also suggest that the PsVs had a high transduction efficiency which, more so than the number of PsVs formed, has been shown to be an important factor when using PsVs for genetic transfer [29]. This shows the importance of not only increasing protein yields for capsid formation, but also investigating techniques to enhance packaging, such as the inclusion of specific DNA sequences on the DNA targeted for encapsidation [66,67]. The PsVs used in PBNAs proved their utility in analysing sera or antibodies for neutralisation, and results from these assays indicated that a number of the antibodies had some neutralising effects. However, only one of the antibodies, a monoclonal antibody B1:A1 which is fully neutralising of native virions [68], was able to completely neutralise the PsVs at both 1:200 and 1:500 dilutions, proving that the *T* = 7 PsVs are highly conformationally similar to their native virion counterparts.

## 4. Limitations and Future Work

Overall, this study was successful in its aims of generating functional BPV PsVs in plants and in progressing research into the plant production of VLP- and PsV-based veterinary vaccines. However, certain limitations still exist, and the following considerations may improve outcomes in future studies. For the development of cheap, viable veterinary vaccines, high protein and particle yields per unit biomass would have to be attained. While significant improvements in protein expression and particle production were achieved in this study, expression of recombinant protein may be further improved by increasing the GC content of genes or using alternative codon-optimisations for L1 and L2 [47,69], using expression vectors with different properties [51,56,70], using silencing suppressors [71], or through targeted protein expression [72]. Particle formation and packaging may be enhanced by exploring the packaging of replicons and linear DNA of various sizes [31,50,62]. Furthermore, the chemiluminescent assay used in this study to measure SEAP expression, while highly sensitive, is also expensive, and while the assay may be adapted to make use of colorimetric substrates, some of the sensitivity may be lost and the assay would have to be performed with (up to 10-fold) higher numbers of PsVs [33]. Additionally, the use of the SEAP reporter protein in this study only allowed us to measure the relative expression levels of the transduced cells, but not to count the number of individually infected cells. The use of alternative reporter proteins such as the commonly used green fluorescent protein (GFP) are therefore attractive alternatives, and may have enabled us to measure expression more cheaply, to identify and quantify individually infected cells by fluorescent microscopy or flow cytometry [33,73], and to estimate the number of transducing units present per volume of PsV stock. As GFP can also be used to visually monitor the expression and accumulation of recombinant protein levels in plants prior to harvesting [73,74,75], the use of this reporter gene should be explored in future studies. 

## 5. Conclusions

Papillomaviruses are generally host-specific, which has complicated the development of HPV vaccines that require proof of efficacy for human clinical trials [76]. Animal PVs, such as BPV, have long been used to elucidate mechanisms by which PVs infect hosts and replicate and induce disease. Animal PVs have also been useful for testing the efficacy and safety of vaccines in animal models or assays [29,44], and in PBNA testing of anti-HPV sera, BPV1 PsVs have specifically been recommended as controls for measuring non-specific neutralising activity [33]. In this study, BPV1 *T* = 1 VLPs and *T* = 7 PsVs were produced in *N. benthamiana* in relatively high amounts. Other studies have shown both *T* = 1 and *T* = 7 BPV VLPs to be highly immunogenic [24,44,47], which suggests that the particles generated in this study could be used as candidate prophylactic and/or therapeutic vaccines against BPV1. In a recent study, PsVs based on animal PVs were successfully used as vehicles for the in vivo delivery of genes to a non-host animal (mice) to induce relatively high levels of gene expression [29]. These findings demonstrate the potential of substituting the PsV vectors of non-host PVs, should prior immunity to specific PV species already exist [29]. Furthermore, as demonstrated in this and previous studies [29,62], techniques for PsV production are largely translatable between PV species and support the prospect of developing a single, scalable, and cheaper plant-based production platform for the production of a wide range of PV species and types. 

## 6. Materials and Methods 

### 6.1. Construct Design and Generation of Recombinant Agrobacterium

Native gene sequences encoding for the capsid proteins of BPV1, L1 and L2, were obtained from the PaVE (Papillomavirus Episteme: https://pave.niaid.nih.gov/ BPV1 Genbank ID: X02346) website [77]. The L1 and L2 genes were codon-optimised for *Nicotiana benthamiana* (with final GC contents of ~43% and ~46%, respectively) and *Nco*I and *Xho*I restriction sites were added for cloning into plant expression vectors. The genes were synthesised and delivered in the pUC57 parent vector (GenScript, Nanjing, China) from which they were subcloned into the binary plant expression vector, pTRAc, which targets expression to the cytoplasm [69], and the self-replicating vector, pRIC3.0 [78]. The resulting constructs were transformed into electrocompetent *Agrobacterium tumefaciens* GV3101::pMP90RK (*Agrobacterium*) by electroporation at 1.8 kV, 25 μF, and 200 Ω. Transformants were selected on LB-enriched agar medium (1.0% tryptone, 0.5% yeast extract, and 1.0% NaCl, pH 7.0) supplemented with 50 µg/mL carbenicillin, 50 µg/mL rifampicin, and 30 µg/mL kanamycin (LB_rkc_). Recombinant clones were identified by PCR using vector-specific primers (fw: 5′-CATTTCATTTGGAGAGGACACG-3′ and rev: 5′-GAACTACTCACACATTATTCTGG-3′) and confirmed by sequencing (Macrogen, Amsterdam, Netherlands) to ensure sequence fidelity. Empty pRIC3.0 and pTRAc vectors were also transformed into *Agrobacterium* for use as negative controls.

### 6.2. Transient Expression of BPV1 VLPs in N. benthamiana

Expression conditions conducive to the highest recombinant protein accumulation were determined by small-scale expression studies, in which different cell concentrations (OD_600_) of *Agrobacterium* were used for infiltration, and time-trials were conducted by harvesting biomass at different days post infiltration (dpi). The conditions established in the small-scale expression studies were used for large-scale infiltrations for the production of L1 and L1/L2 VLPs. *Agrobacterium* cultures of the L1 and L2 constructs and empty vector negative controls of pRIC3.0 and pTRAc were grown for two days at 28 °C with 120 rpm agitation in an LBB medium (0.25% tryptone, 1.25% yeast extract, 0.50% NaCl, 10 mM MES (morpholinoethanesulfuric acid), and 1.50% agar, pH 5.6), supplemented with 50 µg/mL carbenicillin, 50 µg/mL rifampicin, and 30 µg/mL kanamycin (LBB_rck_), with the final overnight culture free of rifampicin. The overnight cultures were diluted to optimal concentrations in infiltration medium (10 mM MES, 10 mM MgCl_2_, 3% sucrose, and 200 µM acetosyringone, pH 5.6) and incubated at room temperature for 2 h to allow *vir* genes to be induced by the acetosyringone. Infiltrations were performed by submerging the leaves of 6–8-week-old *N. benthamiana* plants into bacterial suspensions and applying a vacuum pressure of −95 kPa for ~20 s, before releasing the pressure. For each construct or construct pair, 10–15 plants were infiltrated and incubated at 22 °C for 5 days under 16 h: 8 h, light: dark cycles, after which biomass was harvested and processed.

### 6.3. Transient Expression of BPV1 PsVs in N. benthamiana 

BPV1 PsV expression was based on conditions established for HPV16 PsV production [50]. The pseudogenome, pRIC3-mSEAP, used in both this and the aforementioned study, consisted of the self-replicating plant expression vector, pRIC3.0, which was modified to include a mammalian expression cassette encoding for the secreted embryonic alkaline phosphatase (SEAP) reporter gene. Autonomous replication of this construct yields circular replicons of 4.8 kb, which is within the 8 kb size limit capable of being packaged by PV capsid proteins [50,69]. For large scale expression of PsVs, cultures of recombinant *Agrobacterium* containing pTRAc-L1, pTRAc-L2, and pRIC3-mSEAP were grown to scale and prepared in infiltration medium to concentrations established for HPV PsV production in plants [50]. The plants were vacuum infiltrated and incubated as previously described, and biomass was harvested 4 days post infiltration (dpi).

### 6.4. Large-Scale Protein Purification of BPV1 VLPs and PsVs

Biomass (40 g/purification) was homogenised in 2× (*v*/*w*) extraction buffer (0.1 M NaOAc/0.5 M NaCl, pH5.2) (NaOAc) supplemented with 1× Complete Mini EDTA-free Protease Inhibitor Cocktail (Roche, Basel, Switzerland), using a T25 digital ULTRA-TURRAX^®^ homogeniser (IKA). Homogenates were incubated for 2 h at 4 °C with shaking, filtered through 4 layers of Miracloth (Calbiochem), and clarified by centrifugation at 10,000× *g* (Beckman Coulter Avanti^®^ J25TI centrifuge) at 4 °C for 15 min. Density gradients of 1 mL 50% and 5 mL 30% *w/v* iodixanol were prepared with extraction buffer and layered beneath the clarified crude extract. Protein was partially purified by ultracentrifugation at 174,500× *g* for 1 h and 15 min at 4 °C, and the 5 mL 30% cushions collected and combined. These samples were dialysed overnight at 4 °C in 14,000-MW cut-off tubing (Sigma #D0405), suspended in 60–100× *v/v* dialysis buffer (1× HSPBS, pH 7.4). Further protein purifications were performed by isopycnic centrifugation on discontinuous iodixanol (OptiPrep^TM^) gradients prepared in 1× HSPBS (1× PBS/0.5 M NaCl, pH 7.4) to final concentrations of 50% (1 mL), 39% (2 mL), 33% (5 mL), and 27% (5 mL) *w/v* iodixanol, which were layered beneath the dialysed samples with a long syringe needle. Ultracentrifugation was performed at 174,500× *g* in a SW 32 Ti Rotor (Beckman) for 3 h and 30 min at 15 °C. Samples were manually fractionated into 1 mL fractions from the bottom of the centrifuge tubes and immediately used for analysis or stored at −80 °C. 

### 6.5. SDS-PAGE and Western Blots

Protein samples were denatured at 95 °C for 10 min in sample application buffer (5× SAB: 2% SDS, 100 mM Tris-Cl (pH 7.5), 2 mM EDTA, 52% glycerol, and 4.3% B-mercaptoethanol) and equal volumes were loaded onto 10% SDS-PAGE gels, alongside the Colour Protein Standard (Broad Range: 11–2545 kDa) (#P7712S, NEB) molecular weight marker, and proteins were separated in a Mini-PROTEAN Tetra Cell (Bio-Rad, Hercules, CA, USA) at 120 V for approximately 80 min. The gels were stained with 1% Coomassie Brilliant Blue stain (Bio-Rad) to determine protein yields and inspect whether the contaminating plant proteins were co-purified with the VLPs, or they were transferred onto nitrocellulose membranes for western blot analysis. Protein transfer to nitrocellulose membranes (Amersham™ Protran^®^, Merck) was performed in the Trans-blot^®^ Semi-dry Transfer Cell at 15 V for 1 h 15 min, with blotting paper soaked with transfer buffer (25 mM Tris, 190 mM glycine, 20% methanol, pH 9.2). Following transfer, the membranes were blocked for 30 min in blocking buffer (1× PBS + 0.1% Tween-20, +5% NFDM (non-fat dairy milk)), after which they were probed with antibodies diluted in blocking buffer. For BPV L1 blots, two primary antibodies were used within this study: (i) a mouse monoclonal antibody (BPV-1/1H8 + CAMVIR) (Abcam #2417), referred to as “Abcam”, which was used at 1:1000 dilution with a goat anti-mouse IgG whole molecule-AP (Sigma #A3562) secondary antibody used at 1:5000; or (ii) a polyclonal rabbit anti-BPV1 (Dako: B0580, Carpinteria, CA), referred to as “Dako”, used at a 1:1000 dilution with a goat anti-rabbit IgG whole molecule-AP (Sigma-Aldrich Sigma #A3687) secondary antibody at 1:5000. The blots were developed with BCIP/NBT solution (Sigma-Aldrich, #B1911) for 1 h in the dark.

### 6.6. Particle Visualisation and Analysis

Transmission electron microscopy (TEM) was used to visualise the purified protein fractions with the highest L1 yields, as determined by western blot analyses. For this, carbon-coated copper grids of mesh size 200 were glow discharged at 25 mA for 30 s using a Model 900 SmartSet Cold Stage Controller (Electron Microscopy Sciences) and placed onto 20 μL samples for 5 min, after which they were washed twice with double-distilled water and negatively stained for 1 min with 2% *w/v* uranyl acetate. The grids were viewed using a Technai 20 transmission electron microscope (FEI) at 27,000–53,000× magnification.

### 6.7. Optimisation of Protein Expression and Purification

Following the successful production of VLPs and PsVs, the expression and purification methods were further optimised. PsVs were used for these analyses as they could be quantitatively assessed by the SEAP assay, and as greater numbers of particles were produced by PsV expression. Purification strategies that were explored included: (i) comparing fresh vs. frozen plant material to see whether losses occur with freezing; (ii) increasing salt content in gradient buffers to enhance particle assembly/stability; and (iii) comparing iodixanol with sucrose gradients. Strategies to enhance the expression of recombinant protein included two methods described in Norkunas et al. (2018) [56]: (i) increasing the acetosyringone levels in the infiltration medium from 200 to 500 µM, and (ii) exposing infiltrated plants to a brief heat shock treatment at 37 °C for 30 min at 2 dpi. A third method, which was based on observations in this study and findings by Buck et al. (2005) [62] and was aimed at enhancing the formation and maturation of PV capsids, was also tested. For this method, plants were incubated for an additional 2 days before their biomass was harvested (6 dpi) to extend the period for expression and assembly/maturation of recombinant protein in planta, prior to harvesting.

### 6.8. Growth and Maintenance of HEK293TT Cell Culture

HEK293TT cells were used for all mammalian cell culture experiments. Cells were cultured in complete Dulbecco’s Modified Eagle Medium (cDMEM) with 1% GlutaMAX (Life Technologies, Carlsbad, CA, USA), which was supplemented with 10% Foetal Bovine Serum (Hyclone™ FBS, Separations), 1% non-essential amino acids (Gibco), Penicillin-Streptomycin (100 units/mL penicillin, 100 μg/mL streptomycin) (Sigma-Aldrich), and 250 μg/mL Hygromycin B (Roche). Cells were incubated at 37 °C in 5% CO_2_ and 95% humidity. Cells were passaged when they were 90% confluent, with a seeding density of 10% (approximately 1 × 10^5^ cells/mL).

### 6.9. SEAP Assay for PsV Infection of Mammalian Cells

In order to use the BPV PsVs to perform vaccine testing in PBNAs, it was first necessary to demonstrate that the PsVs were able to infect mammalian cells and to transfer and express their encapsidated reporter gene to the cells. To this end, HEK293TT cells were trypsinised, resuspended in complete DMEM (cDMEM) medium, and counted using a haemocytometer. Cells were seeded in a Corning^®^ Costar^®^ 24-well plate (Merck) at 1.5 × 10^5^ cells/well, and incubated overnight at 37 °C, 5% CO2. PsV fractions were diluted 1:10 in cDMEM and added to the cells in triplicate at 500 μL/well. The cells were further incubated for 72 h, after which SEAP activity was assayed using the Great EscAPe SEAP Chemiluminescence Kit (Clontech Laboratories, Inc., Mountain View, CA, USA) as per the manufacturer’s instructions, with dilutions and reagents modified to 0.6× the volumes specified (60 uL total reaction volume). SEAP signal was read using a GloMax^®^ 20/20 Luminometer (Promega).

### 6.10. Neutralisation of Plant-Produced BPV1 Pseudovirions by BPV, HPV, and PV Antibodies

To assess the functionality of the plant-produced BPV PsVs for evaluating the protective immunity elicited by vaccines, a PBNA was performed by pre-incubating the purified PsVs with various mono- and polyclonal PV antibodies, before applying them to mammalian cells. The antibodies applied to the BPV VLPs included the mouse monoclonal antibodies: B1:A1 [68], B1:B3, B1:E2, B1:E3, H16:J4 (which recognises a linear neutralising epitope on HPV16 L1 [79]), and the “Abcam” antibody (Abcam, #ab2417), as well as the polyclonal rabbit anti-BPV1 “Dako” antibody, an in-house polyclonal anti-Gardasil antibody, and a PV antibody of unknown origin and specifications. The PV antibody was tested in an ELISA with the BPV VLPs, and was found to have been raised in rabbits. HEK293TT cells were prepared at 1.5 × 10^5^ cells/well in a 24-well plate, as previously described for the SEAP assay, and were incubated overnight at 37 °C, 5% CO_2_, and 95% humidity. The following day, the PsV fraction F9, for which an average SEAP reading of ~1.3 × 10^6^ RLUs had previously been obtained, was diluted to 1:10 in DMEM without antibiotics or serum. The antibodies were applied to the PsVs at a final concentration of 1:200 and were incubated with the PsVs for 1 h at 4 °C before being applied to the cells. Controls of uninfected cells (“cells only”) and cells infected with PsVs not incubated with antibodies (“PsVs only”) were included in the analyses, and all reactions were performed in duplicate. The cells were incubated for a further 72 h, and SEAP reporter gene expression was measured as previously described. This experiment was repeated with antibodies applied at 1:500 dilution.

## Supplementary Materials

The following are available online at https://www.mdpi.com/2076-0817/9/12/996/s1. Figure S1: Transmission electron micrographs of bovine papillomavirus 1 (BPV1) L1-only virus-like particles (VLPs) expressed by pRIC3.0 and pTRAc plant expression vectors; Figure S2: Transmission electron micrographs of bovine papillomavirus 1 (BPV1) L1-only and L1/L2 virus-like particles (VLPs) expressed by pTRAc; Figure S3: Transmission electron micrographs of plant-produced bovine papillomavirus 1 (BPV1) virus-like particle (VLP) and pseudovirion (PsV) purifications; Figure S4: Effect of salt concentration on purifications of plant-produced bovine papillomavirus 1 (BPV1) pseudovirions (PsVs); Figure S5: Bar graph of SEAP assay readings from 1× HSPBS-iodixanol vs. 6× HSPBS-iodixanol purified bovine papillomavirus 1 (BPV1) pseudovirions (PsVs); Figure S6: Expression optimisation of bovine papillomavirus 1 (BPV1) pseudovirions (PsVs).

## References

[1] Campo M.S., Roden R.B.S. (2010). Papillomavirus Prophylactic Vaccines: Established Successes, New Approaches. J. Virol..

[2] Araldi R.P., Muro S., Assaf R., Carvalho R.F.D., Caldas M.A., Carvalho R.D., Souza J.M.D., Magnelli R.F., Grando D., Roperto F.P. (2017). Papillomaviruses: A systematic review. Genet. Mol. Biol..

[3] Bocaneti F., Altamura G., Corteggio A., Velescu E., Roperto F., Borzacchiello G. (2016). Bovine Papillomavirus: New Insights into an Old Disease. Transbound. Emerg. Dis..

[4] Savini F., Gallina L., Prosperi A., Puleio R., Lavazza A., Marco P.D., Tumino S., Moreno A., Lelli D., Guercio A. (2020). Bovine Papillomavirus 1 Gets Out of the Flock: Detection in an Ovine Wart in Sicily. Pathogens.

[5] Roperto S., Russo V., Corrado F., De Falco F., Munday J.S., Roperto F. (2018). Oral fibropapillomatosis and epidermal hyperplasia of the lip in newborn lambs associated with bovine Deltapapillomavirus. Sci. Rep..

[6] Munday J.S. (2014). Papillomaviruses in felids. Vet. J..

[7] Orbell G.M.B., Young S., Munday J.S. (2011). Cutaneous Sarcoids in Captive African Lions Associated With Feline Sarcoid-Associated Papillomavirus Infection. Vet. Pathol..

[8] Williams J.H., van Dyk E., Nel P.J., Lane E., Van Wilpe E., Bengis R.G., de Klerk-Lorist L.M., van Heerden J. (2011). Pathology and immunohistochemistry of papillomavirus-associated cutaneous lesions in Cape mountain zebra, giraffe, sable antelope and African buffalo in South Africa. J. S. Afr. Vet. Assoc..

[9] Van Dyk E., Bosman A.-M., Van Wilpe E., Williams J.H., Bengis R.G., Van Heerden J., Venter E.H. (2011). Detection and characterisation of papillomavirus in skin lesions of giraffe and sable antelope in South Africa. J. S. Afr. Vet. Assoc..

[10] Silvestre O., Orzacchiello G.B., Ava D.N., Ovane G.I., Usso V.R., Ecchio D.V., Usilio F.D.A., Ault E.A.G., Ampo M.S.C., Aciello O.P. (2009). Bovine Papillomavirus Type 1 DNA and E5 Oncoprotein Expression in Water Buffalo Fibropapillomas. Vet. Pathol..

[11] Roperto S., Russo V., Ozkul A., Sepici-dincel A., Maiolino P., Borzacchiello G., Marcus I., Esposito I., Riccardi M.G., Roperto F. (2013). Communication Bovine papillomavirus type 2 infects the urinary bladder of water buffalo (Bubalus bubalis) and plays a crucial role in bubaline urothelial carcinogenesis. J. Gen. Virol..

[12] Pangty K., Singh S., Goswami R., Saikumar G., Somvanshi R. (2010). Detection of BPV-1 and -2 and Quantification of BPV-1 by Real-Time PCR in Cutaneous Warts in Cattle and Buffaloes. Transbound. Emerg. Dis..

[13] Campo M.S. (2002). Animal models of papillomavirus pathogenesis. Virus Res..

[14] Taylor S., Haldorson G. (2013). A review of equine sarcoid. Equine Vet. Educ..

[15] Lunardi M., Alcântara K.D., Arellano A., Rodrigues B. (2013). Bovine Papillomavirus Type 13 DNA in Equine Sarcoids. J. Clin. Microbiol..

[16] Pangty K., Singh S., Pandey A.B., Somvanshi R. (2010). Preliminary binary ethylenimine (BEI) inactivated bovine papillomavirus (BPV) vaccine trial against cutaneous warts in bull calves : A pathological assessment. Braz. J. Vet. Pathol..

[17] Terziev G., Roydev R., Kalkanov I., Borissov I., Dinev I. (2015). Papillomatosis in heifers—Comparative studies on Surgical excision and autogenous vaccine therapies. Trakia J. Sci..

[18] Kale M., Saltik H.S., Hasircioglu S., Yildirim Y., Yavru S., Mamak N., Atli K. (2019). Treatment of Bovine papillomavirus-induced teat warts in a cow by using Podophyllin magistral formula and autologous vaccine applications together. Indian J. Anim. Res..

[19] Rothacker C.C., Boyle A.G., Levine D.G. (2014). Autologous vaccination for the treatment of equine sarcoids: 18 cases (2009–2014). Can. Vet. J..

[20] Liu F., Ge S., Li L., Wu X., Liu Z., Wang Z. (2012). Virus-like particles: Potential veterinary vaccine immunogens. Res. Vet. Sci..

[21] Liu F., Wu X., Li L., Ge S., Liu Z., Wang Z. (2013). Virus-like particles: Promising platforms with characteristics of DIVA for veterinary vaccine design. Comp. Immunol. Microbiol. Infect. Dis..

[22] Kirnbauer R., Chandrachud L.M., O’Neil B.W., Wagner E.R., Grindlay G.J., Armstrong A., McGarvie G.M., Schiller J.T., Lowy D.R., Campo M.S. (1996). Virus-like particles of bovine papillomavirus type 4 in prophylactic and therapeutic immunization. Virology.

[23] Campo M.S. (1997). Vaccination against papillomavirus in cattle. Clin. Dermatol..

[24] Hartl B., Hainisch E.K., Shafti-Keramat S., Kirnbauer R., Corteggio A., Borzacchiello G., Tober R., Kainzbauer C., Pratscher B., Brandt S. (2011). Inoculation of young horses with bovine papillomavirus type 1 virions leads to early infection of PBMCS prior to pseudo-sarcoid formation. J. Gen. Virol..

[25] Kirnbauer R., Booy F., Cheng N., Lowy D.R., Schiller J.T. (1992). Papillomavirus L1 major capsid protein self-assembles into virus-like particles that are highly immunogenic. Proc. Natl. Acad. Sci. USA.

[26] Shafti-Keramat S., Schellenbacher C., Handisurya A., Christensen N., Reininger B., Brandt S., Kirnbauer R. (2009). Bovine papillomavirus type 1 (BPV1) and BPV2 are closely related serotypes. Virology.

[27] Gaukroger J.M., Chandrachud L.M., O’Neil B.W., Grindlay G.J., Knowles G., Campo M.S. (1996). Vaccination of cattle with bovine papillomavirus type 4 L2 elicits the production of virus-neutralizing antibodies. J. Gen. Virol..

[28] Mariz F.C., Jesus A.L.S., Silva M.A.R. (2016). The Challenges Inherent in the Control and Prevention of Bovine Papillomaviruses. Austin J. Genet. Genom. Res..

[29] Bayer L., Gumpel J., Hause G., Muller M., Grunwald T. (2018). Non-human papillomaviruses for gene delivery in vitro and in vivo. PLoS ONE.

[30] Kines R.C., Zarnitsyn V., Johnson T.R., Pang Y.S., Corbett K.S., Nicewonger J.D., Gangopadhyay A., Chen M., Liu J. (2015). Vaccination with Human Papillomavirus Pseudovirus-Encapsidated Plasmids Targeted to Skin Using Microneedles. PLoS ONE.

[31] Cerqueira C., Thompson C.D., Day P.M., Pang Y.-Y.S., Lowy D.R., Schiller J.T. (2017). Efficient Production of Papillomavirus Gene Delivery Vectors in Defined In Vitro Reactions. Mol. Ther. Methods Clin. Dev..

[32] Buck C.B., Pastrana D.V., Lowy D.R., Schiller J.T., Al B.E.T. (2004). Efficient Intracellular Assembly of Papillomaviral Vectors. J. Virol..

[33] Buck C.B., Pastrana D.V., Lowy D.R., Schiller J.T. (2005). Generation of HPV pseudovirions using transfection and their use in neutralization assays. Methods Mol. Med..

[34] Pastrana D.V., Buck C.B., Pang Y.Y.S., Thompson C.D., Castle P.E., FitzGerald P.C., Kjaer S.K., Lowy D.R., Schiller J.T. (2004). Reactivity of human sera in a sensitive, high-throughput pseudovirus-based papillomavirus neutralization assay for HPV16 and HPV18. Virology.

[35] Zhao H., Lin Z.J., Huang S.J., Li J., Liu X.H., Guo M., Zhang J., Xia N.S., Pan H.R., Wu T. (2014). Correlation between ELISA and pseudovirion-based neutralisation assay for detecting antibodies against human papillomavirus acquired by natural infection or by vaccination. Hum. Vaccines Immunother..

[36] Smith J.F., Brownlow M., Brown M., Kowalski R., Esser M.T., Ruiz W., Barr E., Brown D.R., Bryan J.T. (2007). Antibodies from women immunized with Gardasil^®^ cross-neutralize HPV 45 pseudovirions. Hum. Vaccin..

[37] Sehr P., Rubio I., Seitz H., Putzker K., Ribeiro-Müller L., Pawlita M., Müller M. (2013). High-Throughput Pseudovirion-Based Neutralization Assay for Analysis of Natural and Vaccine-Induced Antibodies against Human Papillomaviruses. PLoS ONE.

[38] Leung T.F., Liu A.P.Y., Lim F.S., Thollot F., Oh H.M.L., Lee B.W., Rombo L., Tan N.C., Rouzier R., De Simoni S. (2018). Comparative immunogenicity and safety of human papillomavirus (HPV)-16/18 AS04-adjuvanted vaccine and 4vHPV vaccine administered according to two- or three-dose schedules in girls aged 9–14 years: Results to month 36 from a randomized trial. Vaccine.

[39] Yeager M.D., Aste-Amezaga M., Brown D.R., Martin M.M., Shah M.J., Cook J.C., Christensen N.D., Ackerson C., Lowe R.S., Smith J.F. (2000). Neutralization of human papillomavirus (HPV) pseudovirions: A novel and efficient approach to detect and characterize HPV neutralizing antibodies. Virology.

[40] Sankaranarayanan R., Prabhu P.R., Pawlita M., Gheit T., Bhatla N., Muwonge R., Nene B.M., Esmy P.O., Joshi S., Poli U.R.R. (2016). Immunogenicity and HPV infection after one, two, and three doses of quadrivalent HPV vaccine in girls in India: A multicentre prospective cohort study. Lancet Oncol..

[41] Dessy F.J., Giannini S.L., Bougelet C.A., Kemp T.J., David M.P.M., Poncelet S.M., Pinto L.A., Wettendorff M.A. (2008). Correlation between direct ELISA, single epitope-based inhibition ELISA and pseudovirion-based neutralization assay for measuring anti-HPV-16 and anti-HPV-18 antibody response after vaccination with the AS04-adjuvanted HPV-16/18 cervical cancer vaccine. Hum. Vaccin..

[42] Yin F., Wang Y., Chen N., Jiang D., Qiu Y., Wang Y., Yan M., Chen J., Zhang H., Liu Y. (2017). A novel trivalent HPV 16/18/58 vaccine with anti-HPV 16 and 18 neutralizing antibody responses comparable to those induced by the Gardasil quadrivalent vaccine in rhesus macaque model. Papillomavirus Res..

[43] Folschweiller N., Teixeira J., Joshi S., Goldani L.Z., Supparatpinyo K., Basu P., Chotpitayasunondh T., Chetchotisakd P., Ruxrungtham K., Roteli-Martins C. (2020). Immunogenicity and safety of the AS04-HPV-16/18 and HPV-6/11/16/18 human papillomavirus vaccines in asymptomatic young women living with HIV aged 15–25 years: A phase IV randomized comparative study. EClinicalMedicine.

[44] Hainisch E.K., Abel-reichwald H., Shafti-keramat S., Pratscher B., Corteggio A., Borzacchiello G., Wetzig M., Jindra C., Tichy A., Kirnbauer R. (2017). Potential of a BPV1 L1 VLP vaccine to prevent BPV1- or BPV2- induced pseudo-sarcoid formation and safety and immunogenicity of EcPV2 L1 VLPs in horse. J. Gen. Virol..

[45] Dubey K.K., Luke G.A., Knox C., Kumar P., Pletschke B.I., Singh P.K., Shukla P. (2018). Vaccine and antibody production in plants : Developments and computational tools. Brief. Funct. Genom..

[46] Rybicki E.P. (2014). Plant-based vaccines against viruses. Virol. J..

[47] Love A.J., Chapman S.N., Matic S., Noris E., Lomonossoff G.P., Taliansky M. (2012). In planta production of a candidate vaccine against bovine papillomavirus type 1. Planta.

[48] Zahin M., Joh J., Khanal S., Husk A., Mason H., Warzecha H., Ghim S.J., Miller D.M., Matoba N., Jenson A.B. (2016). Scalable production of HPV16 L1 protein and VLPs from tobacco leaves. PLoS ONE.

[49] Thuenemann E.C., Lenzi P., Love A.J., Taliansky M., Bécares M., Zuñiga S., Enjuanes L., Zahmanova G.G., Minkov I.N., Noris E. (2013). The Use of Transient Expression Systems for the Rapid Production of Virus-like Par- ticles in Plants. Curr. Pharm. Des..

[50] Lamprecht R.L., Kennedy P., Huddy S.M., Bethke S., Hendrikse M., Hitzeroth I.I., Rybicki E.P. (2016). Production of Human papillomavirus pseudovirions in plants and their use in pseudovirion-based neutralisation assays in mammalian cells. Nat. Sci. Rep..

[51] Matić S., Masenga V., Poli A., Rinaldi R., Milne R.G., Vecchiati M., Noris E. (2012). Comparative analysis of recombinant Human Papillomavirus 8L1 production in plants by a variety of expression systems and purification methods. Plant Biotechnol. J..

[52] Baker T.S., Newcomb W.W., Olson N.H., Cowsert L.M., Olson C., Brown J.C. (1991). Structures of bovine and human papillomaviruses. Analysis by cryoelectron microscopy and three-dimensional image reconstruction. Biophys. J..

[53] Belnap D.M., Olson N.H., Cladel N.M., Newcomb W.W., Brown J.C., Kreider J.W., Christensen N.D., Baker T.S. (1996). Conserved features in papillomavirus and polyomavirus capsids. J. Mol. Biol..

[54] Broniarczyk J., Massimi P., Pim D., Marušic B., Myers M.P., Garcea R.L., Banks L. (2019). Phosphorylation of human papillomavirus type 16 L2 contributes to efficient virus infectious entry. J. Virol..

[55] Sweke R. Particle Counter, GitHub, 2017. https://github.com/CorrieGunter/particle_counter.

[56] Norkunas K., Harding R., Dale J., Dugdale B. (2018). Improving agroinfiltration—Based transient gene expression in Nicotiana benthamiana. Plant Methods.

[57] Schiller J.T., Lowy D.R. (2009). Immunogenicity Testing in Human Papillomavirus Virus-Like-Particle Vaccine Trials. J. Infect. Dis..

[58] Fleury M.J.J., Touzé A., de Sanjosé S., Bosch F.X., Klaustermeiyer J., Coursaget P. (2008). Detection of Human Papillomavirus Type 31-Neutralizing Antibodies from Naturally Infected Patients by an Assay Based on Intracellular Assembly of Luciferase-Expressing Pseudovirions. Clin. Vaccine Immunol..

[59] Jiang R.T., Schellenbacher C., Chackerian B., Roden R.B.S. (2016). Progress and prospects for L2-based human papillomavirus vaccines. Expert Rev. Vaccines.

[60] Roden R.B.S., Day P.M., Bronzo B.K., Iv W.H.Y., Yang Y., Lowy D.R., Schiller J.T. (2001). Positively Charged Termini of the L2 Minor Capsid Protein Are Necessary for Papillomavirus Infection. J. Virol..

[61] Jarrett W.F.H., Smith K.T., Neil B.W.O., Gaukroger M., Chandrachud L.M., Al J.E.T. (1991). Studies on Vaccination against Papillomaviruses: Prophylactic and Therapeutic Vaccination with Recombinant Structural Proteins. Virology.

[62] Buck C.B., Thompson C.D., Pang Y.-Y.S., Lowy D.R., Schiller J.T. (2005). Maturation of Papillomavirus Capsids. J. Virol..

[63] Fischer R., Vasilev N., Twyman R.M., Schillberg S. (2015). High-value products from plants: The challenges of process optimization. Curr. Opin. Biotechnol..

[64] Casini G.L., Graham D., Heine D., Garcea R.L., Wu D.T. (2004). In vitro papillomavirus capsid assembly analyzed by light scattering. Virology.

[65] Adams A. (2020). Optimization and Characterisation of Plant Produced Human Papillomavirus Pseudovirions in Nicotiana Benthamiana. Master’s Thesis.

[66] Zhao K.-N., Sun X.-Y., Frazer I.H., Zhou J. (1998). DNA Packaging by L1 and L2 Capsid Proteins of Bovine Papillomavirus Type 1. Virology.

[67] Zhao K.-N., Hengst K., Liu W.-J., Liu Y.H., Liu X.S., McMillan N.A.J., Frazer I.H. (2000). BPV1 E2 Protein Enhances Packaging of Full-Length Plasmid DNA in BPV1 Pseudovirions. Virology.

[68] Christensen N.D., Kreider J.W. (1993). Monoclonal antibody neutralization of BPV-1. Virus Res..

[69] Maclean J., Koekemoer M., Olivier A.J., Stewart D., Hitzeroth I.I., Rademacher T., Fischer R., Williamson A.-L., Rybicki E.P. (2007). Optimization of human papillomavirus type 16 (HPV-16) L1 expression in plants: Comparison of the suitability of different HPV-16 L1 gene variants and different cell-compartment localization. J. Gen. Virol..

[70] Shah K.H., Almaghrabi B., Bohlmann H. (2013). Comparison of Expression Vectors for Transient Expression of Recombinant Proteins in Plants. Plant Mol. Biol. Report..

[71] Reis R.S., Litholdo C.G., Bally J., Roberts T.H., Waterhouse P.M. (2018). A conditional silencing suppression system for transient expression. Sci. Rep..

[72] Yanez R.J.R., Lamprecht R., Granadillo M., Torrens I., Rybicki E.P., Hitzeroth I.I., Biology C., Town C., Africa S., Biology C. (2018). LALF 32-51-E7, a HPV-16 therapeutic vaccine candidate, forms protein body-like structures when expressed in Nicotiana benthamiana leaves. Plant Biotechnol. J..

[73] Soboleski M.R., Oaks J., Halford W.P. (2005). Green fluorescent protein is a quantitative reporter of gene expression in individual eukaryotic cells. FASEB J. Off. Publ. Fed. Am. Soc. Exp. Biol..

[74] Li H.-Y., Chye M.-L. (2009). Use of GFP to investigate expression of plant-derived vaccines. Methods Mol. Biol..

[75] Leffel S.M., Mabon S.A., Stewart C.N. (1997). Applications of Green Fluorescent Protein in Plants. BioTechniques.

[76] Rybicki E.P. (2010). Plant-made vaccines for humans and animals. Plant Biotechnol. J..

[77] NIAID Office of Cyber Infrastructure and Computational Biology (1999). PaVE: Papilloma Virus Genome Database. http://pave.niaid.nih.gov.

[78] Regnard G.L., Halley-Stott R.P., Tanzer F.L., Hitzeroth I.I., Rybicki E.P. (2010). High level protein expression in plants through the use of a novel autonomously replicating geminivirus shuttle vector: Protein expression in plants utilizing BeYDV. Plant Biotechnol. J..

[79] Christensen N.D., Dillner J., Eklund C., Carter J.J., Wipf G.C., Reed C.A., Cladel N.M., Galloway D.A. (1996). Surface Conformational and Linear Epitopes on HPV-16 and HPV-18 L1 Virus-like Particles as Defined by Monoclonal Antibodies. Virology.

