# Supplementary material for: Novel Production of Bovine Papillomavirus Pseudovirions in Tobacco Plants
Source: Pathogens. 2020 Nov 28;9(12):996. doi: 10.3390/pathogens9120996 (PMC7760623; doi:10.3390/pathogens9120996)
Supplement: Supplementary file 1 [file pathogens-09-00996-s001.pdf]

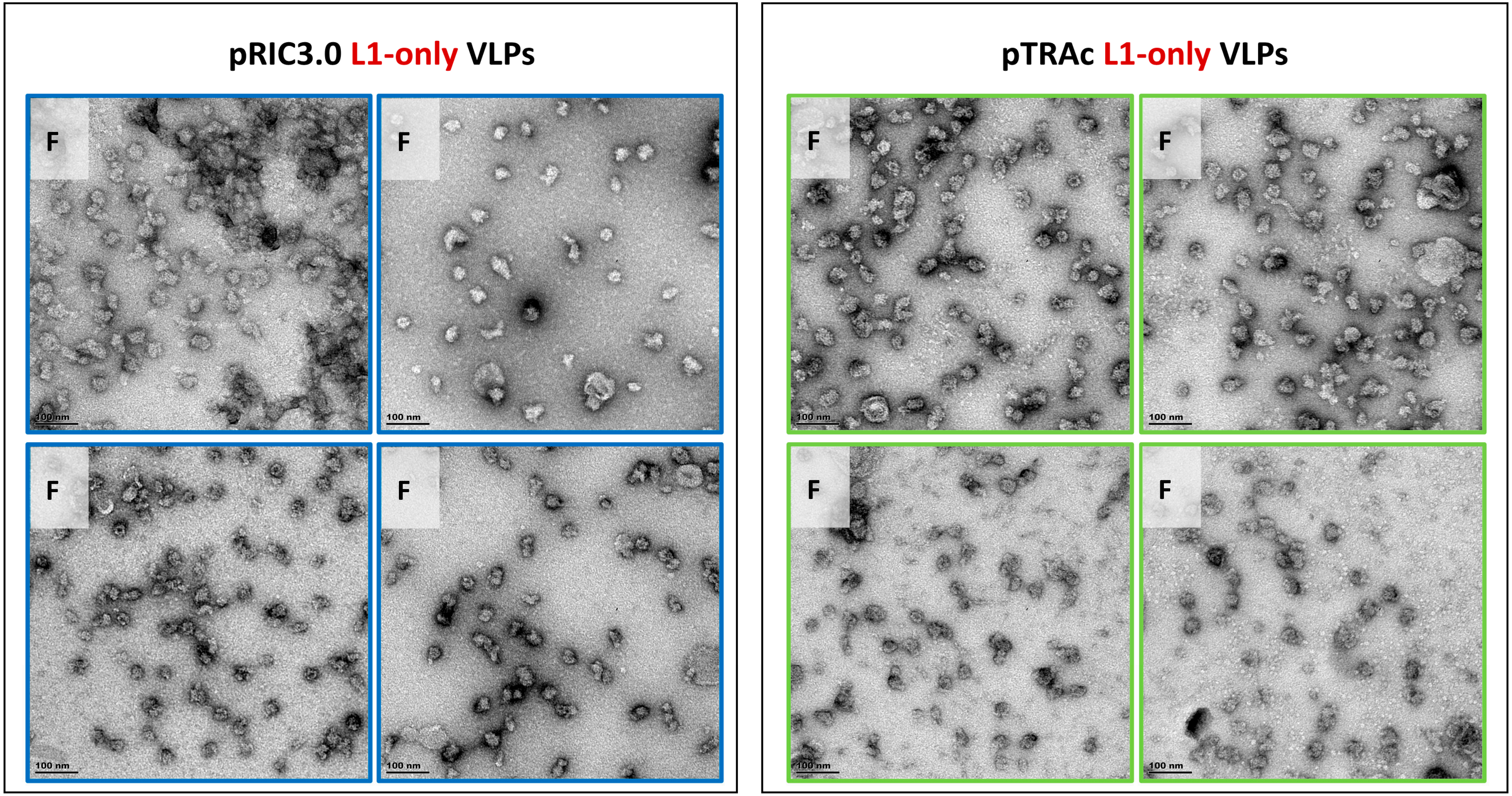

Figure 1. Transmission electron micrographs of bovine papillomavirus 1 (BPV1) L1-only virus-like particles (VLPs) expressed by pRIC3.0 and pTRAc plant expression vectors. Representative protein fractions (F3-F6) of pTRAc- and pRIC3.0-expressed L1, purified on a discontinuous sucrose density gradient. Small *T* = 1 VLPs of 20-30 nm in diameter were observed in both purifications, and pentameric capsomeres of ~10 nm were observed in pTRAc purifications. Scale bars represent 100 nm.

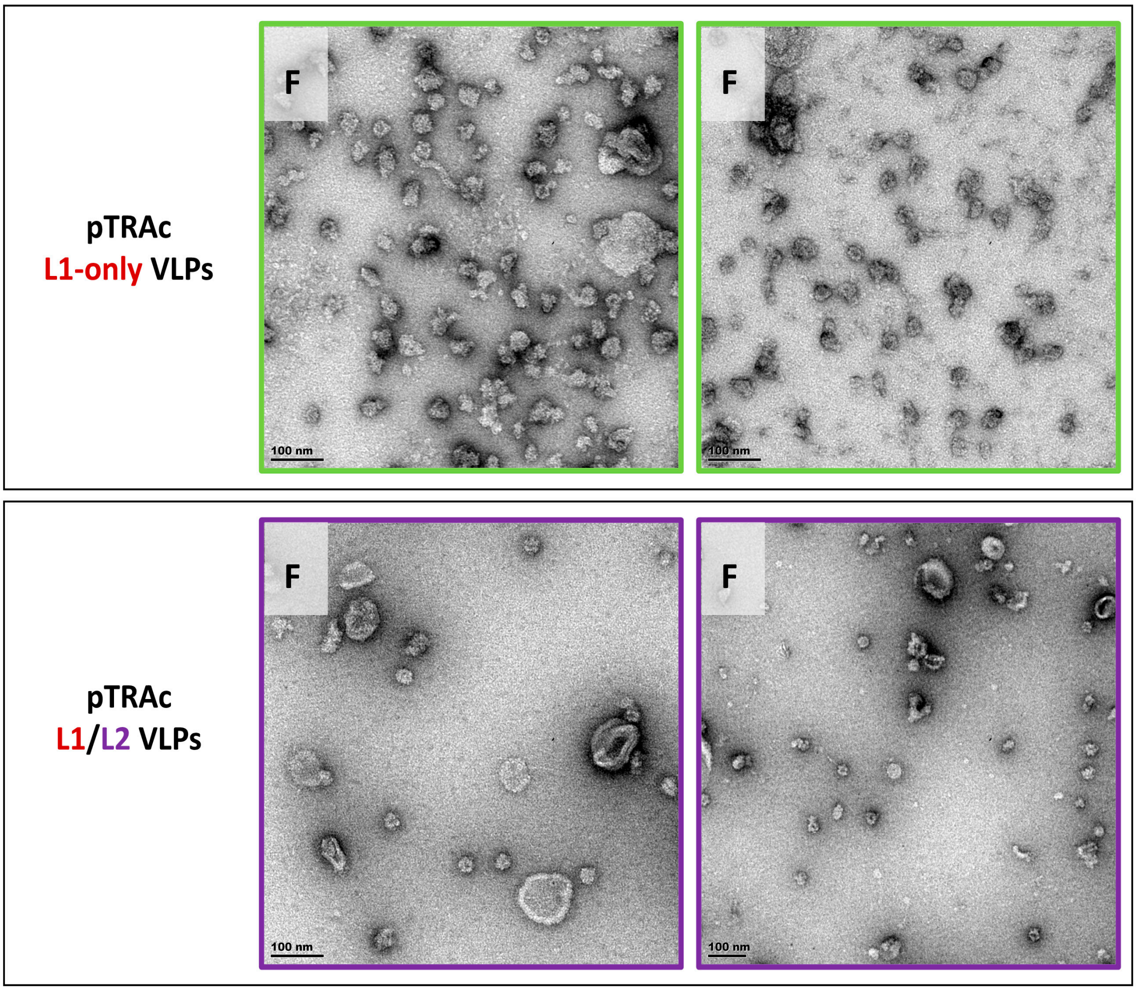

Figure S2. Transmission electron micrographs of bovine papillomavirus 1 (BPV1) L1-only and L1/L2 VLPs expressed by pTRAc. Representative micrographs of protein fractions (F3-F5) obtained from pTRAc-L1 and L1/L2 co-expressions, purified on discontinuous sucrose density gradients. Small, 20-30 nm diameter *T* = 1 VLPs and capsomeres of ~10 nm were observed in both purifications, although fewer particles were present in the L1/L2 purifications. Scale bars represent 100 nm.

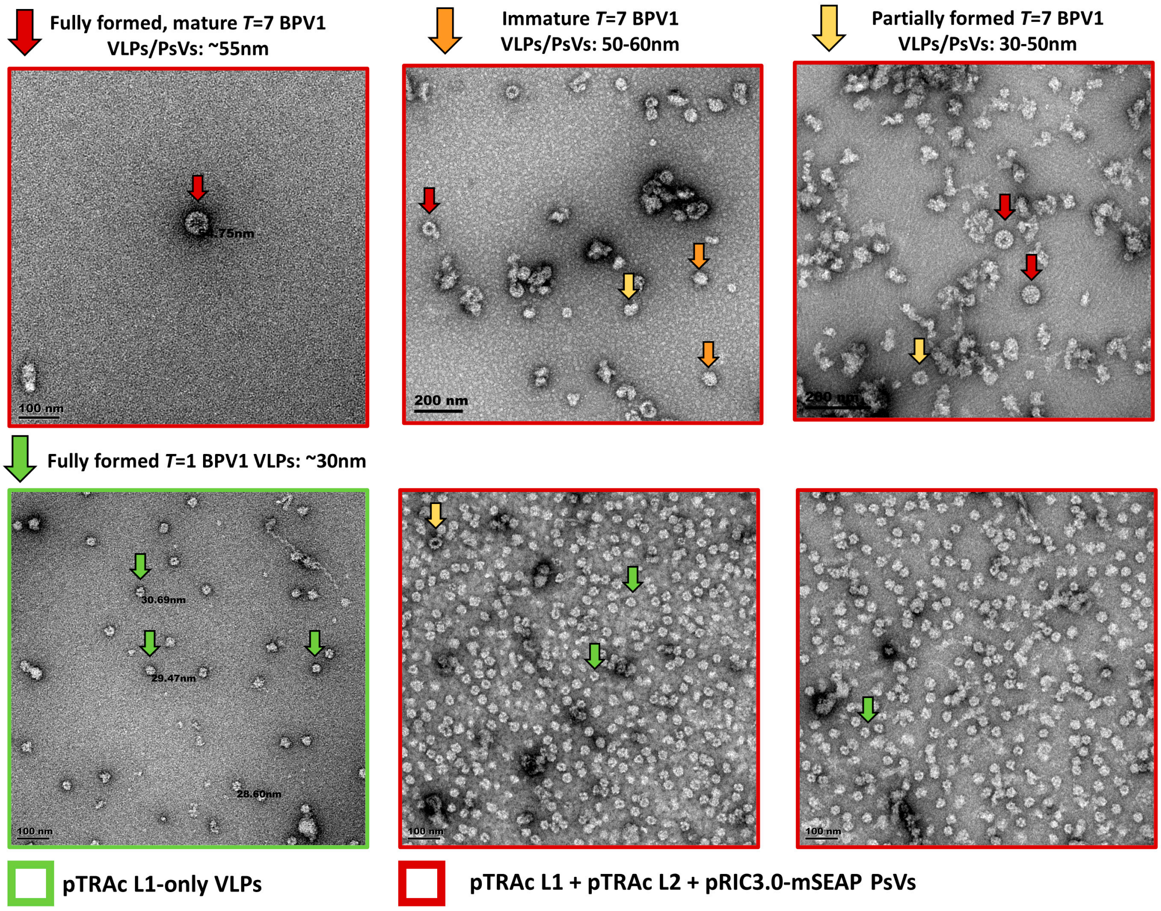

Figure S3. Transmission electron micrographs of plant-produced bovine papillomavirus 1 (BPV1) virus-like particle (VLP) and pseudovirion (PsV) purifications. Electron micrographs displaying the various particle structures observed in VLP and PsV purifications. Fully formed, mature $T = 7$ particles (PsVs and/or VLPs: red arrows) which closely resemble the ~55 nm diameter $T = 7$ structure of native BPV virions were present only in PsV expressions, as well as partially formed $T = 7$ particles of 30-50 nm (yellow arrows), and immature particles of 50-60 nm (orange arrows). In L1 and L1/L2 expressions, only small, $T = 1$ VLPs of 25-30 nm (green arrows) were observed, and many of these particles were also observed in the upper (least dense) fractions of PsV purifications. Scale bars are indicated in each micrograph.

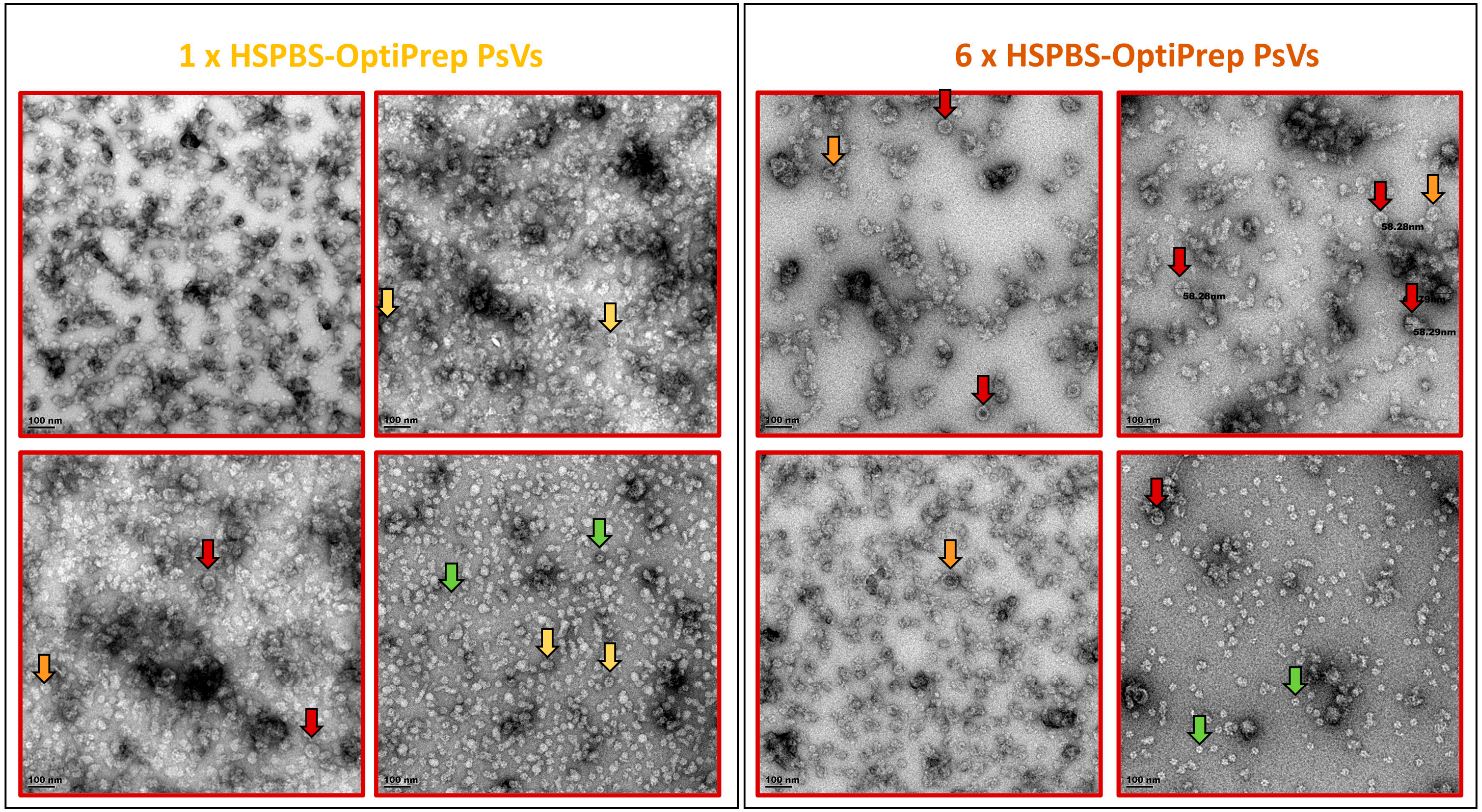

Figure S4. Effect of salt concentration on purifications of plant-produced bovine papillomavirus 1 (BPV1) pseudovirions (PsVs). Electron micrographs of BPV1 PsV proteins purified on discontinuous iodixanol (OptiPrep™) density gradients, prepared with either a 1× HSPBS (1× PBS + 0.5M NaCl) or a 6× HSPBS (6× PBS + 3M NaCl) buffer. In both purifications, *T* = 7 particles (PsVs and/or VLPs: red arrows) resembling native BPV virions were present, as were high numbers of small, 25-30 nm diameter *T* = 1 VLPs (green arrows). However, there appeared to be fewer of the mature, *T* = 7 particles present in the 1× HSPBS purifications, and more loose aggregates and background. In the 6× HSPBS purification, the particles appear to be better separated and concentrated according to their sizes. In both purifications, partially formed *T* = 7 particles of 30-50 nm (yellow arrows) and immature particles of 50-60 nm (orange arrows) were also present. Scale bars represent 100 nm.

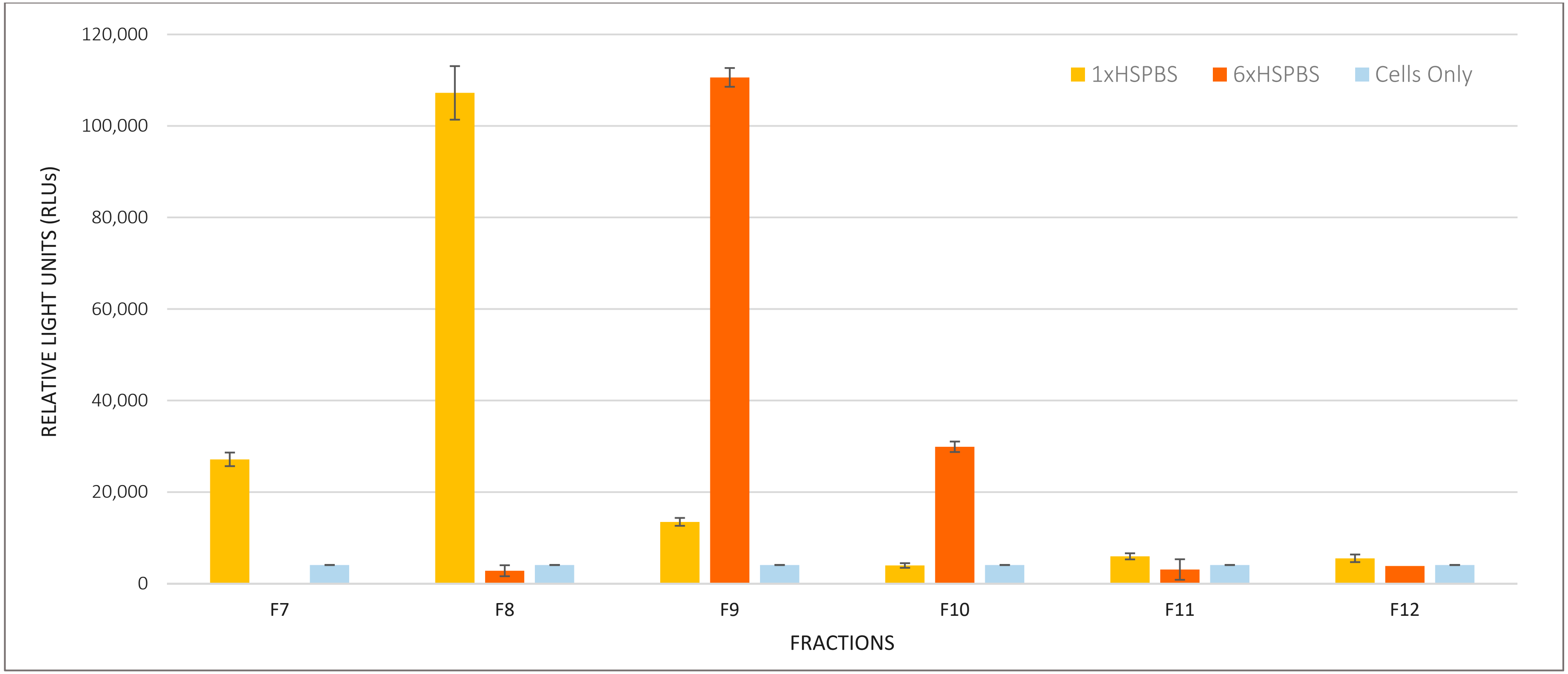

Figure S5. Bar graph of SEAP assay readings from 1× HSPBS-iodixanol vs. 6× HSPBS- iodixanol purified bovine papillomavirus 1 (BPV1) pseudovirions (PsVs). A comparison between the relative SEAP readings (RLUs) of cells, which had been transfected with proteins purified on iodixanol (OptiPrep™) gradients prepared either with 1× HSPBS (yellow) or 6× HSPBS (orange) solutions. The protein fractions applied to cells were those with the highest L1 levels, as identified by western blot. SEAP readings were also compared to a cells-only control (grey), to which no proteins had been applied. The results indicated that 1× HSPBS had slightly higher (5%) cumulative readings, but also showed that the PsVs in the 6× HSPBS purification are concentrated into fewer fractions.

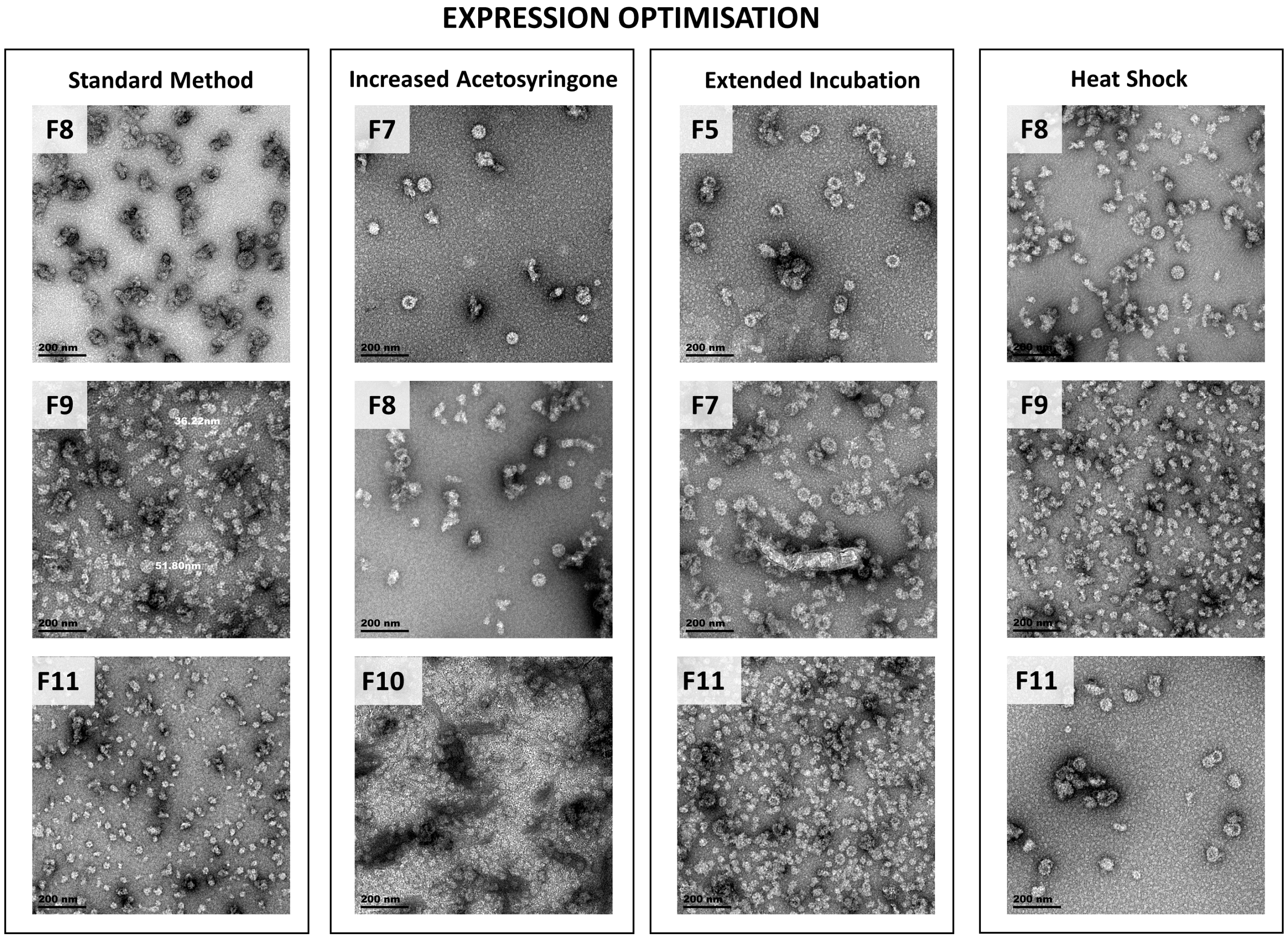

Figure S6. Expression optimisation of bovine papillomavirus 1 (BPV1) pseudovirions (PsVs). Transmission electron micrographs representing fractions of purified proteins from PsVs expressed under the following conditions: Standard protocol, as established for HPV16 PsV expression; Increased acetosyringone (500 μM); Extended in planta incubation (harvested 6 dpi); Heat shock treatment (37 °C). Magnification was performed at 53,000x and scale bars represent 200 nm.
